# Analysis of the Cultured Meat Production System in Function of Its Environmental Footprint: Current Status, Gaps and Recommendations

**DOI:** 10.3390/foods10122941

**Published:** 2021-11-30

**Authors:** María Ignacia Rodríguez Escobar, Erasmo Cadena, Trang T. Nhu, Margot Cooreman-Algoed, Stefaan De Smet, Jo Dewulf

**Affiliations:** 1 Research Group Sustainable Systems Engineering (STEN), Department of Green Chemistry and Technology, Faculty of Bioscience Engineering, Ghent University, Coupure Links 653, 9000 Ghent, Belgium; erasmo.cadenamartinez@ugent.be (E.C.); trang.nhuthuy@ugent.be (T.T.N.); margot.cooreman@ugent.be (M.C.-A.); jo.dewulf@ugent.be (J.D.); 2 Laboratory for Animal Nutrition and Animal Product Quality, Department of Animal Sciences and Aquatic Ecology, Faculty of Bioscience Engineering, Ghent University, Coupure Links 653, 9000 Ghent, Belgium; stefaan.desmet@ugent.be

**Keywords:** life-cycle assessment, environmental sustainability assessment, prospective life-cycle assessment, environmental impact, sustainability, cultured meat, conventional meat

## Abstract

Cultured meat has been presented as an environmentally friendlier option to conventional meat, but due to the limited data, the studies related to its performance are scarce and based on hypothetical production processes. This work provides a short literature review of the published environmental assessments of cultured meat. The main findings of this critical analysis showed that the lack of real data related to cultured meat decreased the level of accuracy of each study. The missing environmental profile of the process itself, including the proliferation and differentiation phases in bioreactors, along with key ingredients such as growth factors and other recombinant proteins, increase the difficulty of achieving reliable conclusions. In order to bridge the highlighted gaps, a complete production system is modelled and analysed from an engineering and life-cycle perspective. Furthermore, an overview of the supply chains of different products used in the process is provided, together with recommendations on how they should be considered in future life-cycle assessments. In essence, this work provides a structured pathway for upcoming consistent environmental assessments in this field, with the objective of setting the basis to understand the potential of cultured meat.

## 1. Introduction

Global demand for livestock products such as meat, milk and eggs, has been and will keep growing for several reasons, including rising incomes and growing populations [1,2]. Food production will therefore have to rise to meet this growing demand [3]. This does not come without concerns, since meat production and consumption have been negatively associated with human health and the environment [4,5,6]. Approximately 80% of the global greenhouse gas (GHG) emissions from food supply chains can be attributed to livestock production, and therefore, efforts to reduce these negative impacts will likely include large-scale dietary changes [7].

About 27% of the total GHG emissions attributed to the livestock sector correspond to carbon dioxide (CO_2_), mainly coming from land-use change and the use of fossil fuels. A similar proportion (29%) is a consequence of the use of fertilisers on feed-crop fields and manure. Finally, as a result of enteric fermentation, manure and rice feed, around 44% of the emissions from this sector are in the form of methane (CH_4_) [8], which is in itself a more potent GHG than the commonly emitted CO_2_ [9]. The global warming potential value in a 100-year time horizon for CH_4_, is 28 (relative to a value of 1 for CO_2_) [10]. Therefore, strong, sustained reductions of CH_4_ can ameliorate the air quality and limit the warming effect it has on the planet [11]. Furthermore, the water footprint related to meat from beef (15,400 m^3^ ton^−1^ as a global average) is significantly larger than the one of sheep (10,400 m^3^ ton^−1^), pig (6000 m^3^ ton^−1^), goat (5500 m^3^ ton^−1^) and chicken (4300 m^3^ ton^−1^) [12]. Health risks related to meat consumption have been highlighted as a concern from the consumers’ side, mainly due to increased additives, hormones, cholesterol levels [13] and a higher risk of developing certain types of cancer, like colorectal cancer [14]. Animal husbandry has also been under scrutiny for ethical reasons. Though the knowledge of consumers regarding farming and animal welfare issues is somewhat low [15,16], the public has regarded these production systems as unacceptable, worrying about the welfare of animals used [17].

Awareness has increased regarding the mentioned issues, which has resulted in a higher popularity of vegan, vegetarian and flexitarian diets [18,19,20]. Consequently, other protein sources have become more common. Plant-based options, such as soy-based protein, legume-based protein and cereal-based protein, are some of the alternatives to conventional meat [21]. Insect-based foods, popular in countries from South and East Asia, Africa, South and Central America and Oceania [22] are another option that provides high protein content. However, it has been shown that the “typical” meat lover in the western world will unlikely consider including insects in their diet [23]. Cultured meat, commonly referred to as “in vitro meat”, “cell-based meat” and “clean meat”, is a product that aims to replicate conventional meat, through (stem) cells and tissue culture [24]. This technology got notoriety after a cultured beef hamburger was tasted on August 5 of 2013, in London [25].

It has been suggested that cultured meat has the potential to significantly reduce the environmental impacts related to meat production, more specifically regarding GHG emissions, land use and water use [26]. The systems to produce cultured meat can be designed to be more efficient regarding the input-to-output ratio than biological ones, avoiding not only direct animal emissions, but also those associated with the production process [27]. However, these results might come at the expense of increased energy use during the process, as key biological functions are in fact replaced by industrial equivalents [28]. Cultured meat could bring improvements for animal welfare [29] and experts have claimed that it could be safer than conventional meat. The argument behind this is that cultured meat will be produced in conditions that are fully under control, without any other organism and in no contact with the external world, decreasing the chances of getting intestinal pathogens such as *E. coli* or *Salmonella* [30]. However, as the authors state, it could be argued that the in vitro process will never be perfectly controlled, and therefore, unexpected issues might occur.

To prove whether cultured meat has the potential to reduce the environmental impacts related to meat production, tools such as life-cycle assessment (LCA) can be of use. This technique allows the assessment of the impacts related to all the stages of a product’s life cycle, including the extraction of raw materials, processing, manufacturing, distribution, and use [31]. This method is used in compliance with international standards (ISO 14040) and consists of four main stages. First, goal and scope, where the part of the product’s life cycle that will be included in the assessment, is defined, as well as the functional unit [31]. Second, the inventory analysis, in which all the materials and energy flows within the system under assessment are described [31]. The third step is the life-cycle impact assessment (LCIA). Here, through mathematical calculations, the boundaries and data are applied to what is being investigated [32]. Different LCIA methods exist to aggregate the inventory data for emissions and resource consumptions and convert them into several midpoint and endpoint impact categories [32]. Common LCIA methods include ecological footprint, cumulated energy demand, CML, eco-indicator, IMPACT 2002+, USEtox 2.01, EDIP 2003, IMPACT world+, ReCiPe, ILCD 2011, TRACI 2.1, LC-Impact and ecological scarcity, among others. A review of LCIA methods is documented in Wu and Su (2020) [32]. The interpretation of the impact assessment results takes place in stage four [31].

Because the large-scale production of cultured meat has yet to exist, only a limited number of studies on environmental issues have been carried out. Furthermore, most of these studies rely on hypothetical production processes and assumptions [33]. Based on this, the research question of the current study arose: from an engineering and life-cycle perspectives, what are the main gaps that should be addressed to have a complete environmental assessment of cultured meat in the future? To fill these gaps, a critical analysis of the published life-cycle assessments of cultured meat is presented, in which the key missing ingredients and processes are highlighted. A proposal for a complete production process of cultured meat has been built upon previous research in order to provide a structured pathway for upcoming LCA studies in this field.

## 2. Materials and Methods

### 2.1. Research Methodology

The research to find LCA-related studies conducted about cultured meat was carried out from November 2020 until June 2021, using several search engines: Scopus, Web of Science (WoS) and Google Scholar. First, search terms were defined and included cultured meat, cultivated meat, in vitro meat, lab-grown meat, fake meat, Frankenstein meat, cell-based meat, environmental impact, sustainability, life-cycle assessment, climate impact. Several combinations of these terms were used such as Cultured meat (and synonyms) AND life cycle assessment, Cultured meat (and synonyms) AND sustainability, Cultured meat (and synonyms) AND climate impact, Cultured meat (and synonyms) AND environment. Secondly, the search was refined in each engine, where only peer-reviewed journals were considered. Only one life-cycle assessment (Tuomisto and Teixeira de Mattos, 2011) was found in Web of Science [26]. In Scopus, two of the life-cycle assessments of cultured meat considered for this study were found (Mattick et al., 2015; Tuomisto and Teixeira de Mattos, 2011) [26,28]. Finally, all of them, including the above-mentioned ones, were found in Google Scholar [26,28,34,35]. It is concluded that very few life-cycle assessments of cultured meat have been published and that the LCAs related to it have been prospective (also called anticipatory or ex-ante LCAs). A prospective LCA is used when the emerging technology studied is modelled towards a future, developed phase, despite being in an early phase of development [36]. To date, the more robust inventory, where primary data was collected from relevant companies, was published in a report by CE Delft, described in the following section [37].

### 2.2. Selected Studies

Out of several peer-reviewed publications that touched upon the subject of environmental sustainability related to cultured meat, a limited number performed LCAs. Some reports have been published in grey literature, such as the proceedings of the 9th International Conference on Life Cycle Assessment in the Agri-Food Sector called “Environmental impacts of cultured meat: alternative production scenarios” by Tuomisto et al. (2014) [35] and an LCA of cultured meat published by the research and consultancy organisation CE Delft, for GAIA and the Good Food Institute [37]. In the present article, the focus lies on peer-reviewed publications and the conference proceeding, since they meet the level of detail required for this type of analysis.

## 3. Results

Table 1 shows the main parameters considered for each of the studies under investigation, which all modelled the production of cultured meat.

Table 2 provides an overview of the main gaps identified in each study. Details will be given in the description of each assessment and their results, and recommendations on how they should be considered for future LCAs can be found in Section 4.

The first published study was presented by Tuomisto and Teixeira de Mattos (2011) [26]. The goal was to carry out an LCA to estimate the potential environmental impacts related to an industrial-scale production of a minced-beef type of cultured meat. They compared three locations: Spain, California (USA) and Thailand. Cell source was not specified, as it was stated that the species only affect which type of growth factors are used and do not change the environmental impacts of the process [26]. The impact indicators considered in this attributional LCA included energy use, GHG emissions, land use and water use to produce 1000 kg of cultured meat (Table 1). The product was compared to conventionally produced beef, sheep, pork and poultry meat, based on the edible weight of the animal.

The study had a cradle-to-gate approach, including all major processes from input production up until the factory gate. As shown in Table 1, cyanobacteria hydrolysate was assumed to be the source of nutrients and energy for the cells. The concrete open-pond cultivation of the cyanobacteria was included in the system boundaries. After being harvested, the cyanobacteria go through a sterilisation and hydrolysis process to break down the cells [26]. However, at hydrolysis, only 50% of the cyanobacteria biomass was assumed to be suitable for muscle cell cultivation and therefore allocated to cultured meat, on a mass-basis. The remaining biomass was assumed to be used as raw material for anaerobic digestion (20%) and food supplements (80%). The biomass going into anaerobic digestion was allocated to cultured meat on a mass-basis, however, not the energy produced by it. Growth factors were produced by engineered *Escherichia coli* bacteria but its manufacturing, along with the one of vitamins, was not included in the calculations. The bioreactor considered for this study was a stainless-steel cylinder stirred-tank bioreactor and its production was included, as shown in Table 2.

A Monte Carlo analysis was used for the sensitivity analysis, simulating 50,000 replications with random input values. The parameters changed were: 40% increase in the cyanobacteria yield, no use of fertilisers and transportation increased by 100 km. In terms of energy, these changes included energy input for sterilisation increased by 50%, the energy input for the steel production increased by 50%, the energy input for the aeration increased by 50% and energy input for the rotation increased by 50%. They also assessed what happened when 100% of the initial cyanobacteria was allocated to cultured meat, when 50% of the initial cyanobacteria was allocated to cultured meat, when 50% was allocated to side products and finally, the use of freshwater for cyanobacteria production.

**Figure 1:**
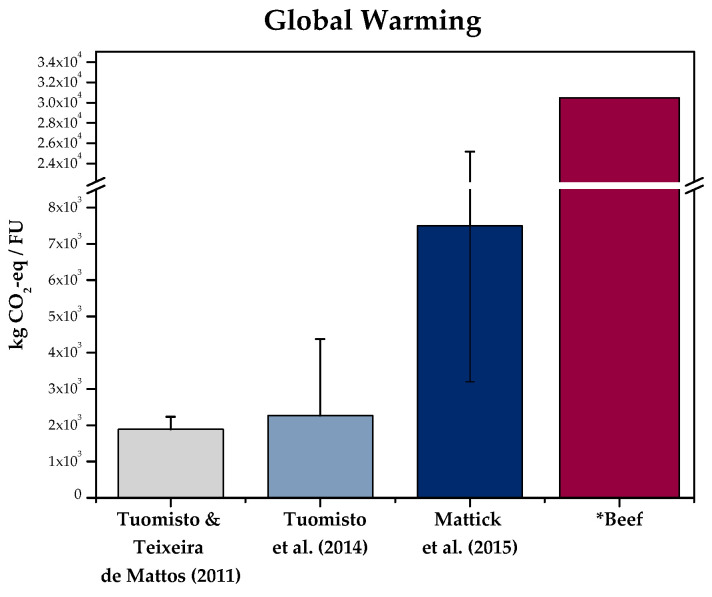
Global warming expressed in kg CO_2_-eq per 1000 kg of cultured meat, per study. *Beef reference was based on the results indicated by Mattick et al. (2015).

The global warming of the hypothetical cultured meat production plant was reported in terms of GHG emissions, measured in kg CO_2_-eq per 1000 kg of cultured meat (see Figure 1). The best-case scenario was the one located in Thailand, where the total emissions amounted to 1891 kg CO_2_-eq FU^−1^. As the authors explained, this was because Thailand had the lowest primary energy requirements to produce electricity. The plant located in Spain emitted 1896 kg CO_2_-eq FU^−1^, and finally, the worst-case scenario was the one located in California, where 2235 kg CO_2_-eq FU^−1^ were reported. California had a high proportion of coal in the electricity mix used in the assessment. In terms of the energy consumption of the whole system, the calculations showed that the best-case scenario was the one located in Thailand, where the energy consumption resulted in 25.2 GJ FU^−1^. The next best scenario would be the plant located in Spain, where the primary energy consumption was up to 31.7 GJ FU^−1^, and the worst-case scenario resulted in the consumption of 31.8 GJ FU^−1^, which is the production plant located in California. The land-use requirements for this study, which included the cultivation of cyanobacteria, resulted in 189 m^2^ FU^−1^ if the plant was in Thailand, 225 m^2^ FU^−1^ in California and finally, 232 m^2^ FU^−1^ in Spain. The energy used to produce cultured meat was reported as significantly lower than for beef, sheep and pork, but not for poultry. In terms of GHG emissions, land use and water use, cultured meat scored better than all the other meat-based products.

Alternative production scenarios were assessed by Tuomisto et al. (2014), in which the main goal was to amend the 2011 study by replacing the cyanobacteria-based media with plant-based media and assessing a different type of bioreactor to produce cultured meat [35]. The production plant was assumed to be in Spain. The estimated environmental impacts resulting from this attributional LCA included energy use, GHG emissions, land use and water use. These impacts were compared to conventionally produced meat (beef, sheep, pork and poultry) and other plant-based protein sources (pulses), on a weight basis. As indicated in Table 1, the functional unit considered for this study was 1000 kg of cultured meat with a dry matter content of 30% and protein content of 19% of the mass.

The system boundaries followed a cradle-to-gate approach, including the production of inputs and fuels, feedstock and the cultivation of muscle cells [35]. Again, cell collection was not included in the process, as shown in Table 2. The medium was assumed to be made of a plant-based energy and nutrient source supplemented with growth factors and vitamins, which were not included in the calculations either (Table 2). Three cultivation mediums were compared in this study: wheat, corn and cyanobacteria hydrolysate, for which the details were taken from Tuomisto and Teixeira de Mattos (2011) [26]. The data collected for wheat and corn production was taken from Williams et al. (2006) and the main assumption for this was that 2 kg of corn or wheat can stimulate the production of 1 kg of cultured meat [38]. It is indicated by the authors that this is most likely an overestimation. The feedstock is again sterilised and hydrolysed. The land required to cultivate the feedstock was included in the study. An economic allocation was chosen as a method to allocate the impacts between the non-edible and edible parts of the animal. Non-edible parts of the animal represent around 10% of the market value, and because of this, 90% of the impacts were allocated to the edible part [35]. Even though they did not specify the species studied, if cattle were the focus, typically edible components besides the meat are liver, tongue, kidneys and others, while non-edible products include skin, hair, horns, bones, feet and more. Differences with the previous study included a change in the bioreactor, namely using a hollow fibre bioreactor to replicate the capillary system found in most tissues [35]. The bioreactor’s production was included in the system boundaries. However, it was this time made of stainless steel and glass wool. Besides analysing the environmental impacts related to three different feed sources, the authors also took into consideration some other variables, including a best-case and worst-case scenario for differences in cell viability, cell yield and starting population.

In terms of global warming, when assessing the best-case scenario using cyanobacteria as feedstock, the plant would emit 2268 kg CO_2_-eq FU^−1^. On the other hand, the worst-case scenario was reported when using wheat as feedstock, with emissions that were as high as 4379 kg CO_2_-eq FU^−1^. Other results included the primary energy consumption, which in the best-case scenario when using corn as a feedstock, resulted in 34.5 GJ FU^−1^. The worst-case scenario when using cyanobacteria had the highest energy consumption, which amounted to 60.9 GJ FU^−1^. The hypothetical plant would need, in the best case, 0.046 ha FU^−1^ when using cyanobacteria as a feedstock and, in the worst-case scenario, 0.282 ha FU^−1^, when using corn. Results showed that, compared to beef, energy inputs were at this time comparable to the ones from beef production, which can be explained by the inclusion of the energy requirements of the bioreactor. Furthermore, in terms of GHG emissions and land use, cultured meat scored lower than the conventional counterparts. Finally, water footprint scored at a comparable level as poultry.

Smetana et al. (2015) performed a comparative analysis of the environmental impacts of several types of meat and their alternatives, including chicken, dairy-based, lab-grown, insect-based, gluten-based, soy-meal-based and mycoprotein-based sources of protein [34]. The data for the production of cultured meat was based on the work performed by Tuomisto and Teixeira de Mattos (2011) [26]. The functional unit selected was 1 kg of ready-to-eat product, with alternative functional units considered in the sensitivity analysis. ReCiPe V1.08 was used as the characterisation method because it performs detailed analysis that includes several impact factors such as climate change, ozone layer depletion, human toxicity, fossil fuel depletion, land use and others. Moreover, IMPACT 2002+ was used as a characterisation method in the sensitivity analysis to compare the results and assess the reliability of the main study.

This study took a cradle-to-plate approach, considering the supply chain from raw material extraction to the consumer’s product use. The products were assembled and transported to a supermarket where they would be cooled down in a cooling counter. It was assumed that the products travelled a distance of 10 km to the customers and were fried with the use of an electric stove [34]. Parameters included in the sensitivity analysis were the equivalent of 3.75 MJ energy content of fried chicken lean meat and 0.3 kg of digested dry matter protein content (1.25 kg of cultured meat).

Regarding GHG emissions, indicated as climate change, Smetana et al. (2015) reported a range between 23.9 and 24.6 kg CO_2_-eq FU^−1^, with cultured meat scoring worse than any other compared product [34]. The energy use amounted to a range between 290 and 373 MJ FU^−1^ and the land occupation varied from 0.39 and 0.77 m^2^ FU^−1^, this value being the highest of all products studied. Compared to other meat products, cultured meat scored worst in almost all categories, except in agricultural land occupation, where the highest impacts came from gluten-based products, and terrestrial and freshwater ecotoxicity, where chicken scored worst. In endpoint categories, cultured meat was also responsible for the highest contribution to human health, resources availability and ecosystem quality. In all those categories, insect-based and soy-meal-based meat substitutes had the lowest impacts.

The most recent published prospective life-cycle analysis of cultured meat production was carried out by Mattick et al. (2015), in which the goal was to model and assess a large-scale biomass cultivation facility in the United States [28]. The functional unit in this study was 1 kg of edible Chinese hamster ovary (CHO) biomass and the correspondent impact categories evaluated included global warming, eutrophication potential and land use. The impacts were then compared to those of beef, pork, poultry and to the results presented in Tuomisto and Teixeira de Mattos (2011) [26].

Mattick et al. (2015) took a cradle-to-gate approach and modelled the production in two phases: proliferation and differentiation [28]. The cultured media was assumed to be a serum-free medium supplemented with soy hydrolysates, and its production process was almost entirely considered, including amino acids production, vitamins, minerals, and basal media formulation. However, growth factors’ production was excluded from the system boundaries (Table 2). The environmental impacts of the inputs and their by-products were allocated based on a gross chemical energy basis. Each phase took place in a stirred tank bioreactor, of which the equipment production was not included within the system boundaries. Scaffold production was included in the LCA, in the form of microcarrier beads made from corn starch. A Monte Carlo analysis was used to perform a sensitivity analysis, wherein the modified factors included facility size, facility energy consumption, cell growth rate, minimum cell density, maximum cell density, mass increase during differentiation, crop yield for corn and crop yield for soybeans.

The results of this life-cycle assessment showed that the total global warming of the production plant under study was around 7.5 kg CO_2_-eq FU^−1^. Furthermore, the range of impact based on the sensitivity analysis went from approximately 3.2 to 25.2 kg CO_2_-eq FU^−1^. Significantly higher results compared to the previous studies were reported in terms of energy consumption, with a total of 106 MJ FU^−1^, including values that ranged from approximately 50 to 355 MJ FU^−1^, in the sensitivity analysis. A total of 5.5 m^2^ FU^−1^ of land would be required for the modelled production process. The production of cultured meat resulted in higher industrial use than livestock production. The authors indicate that cultured meat could have lower amounts of GHG emissions than beef but larger amounts than pork or poultry.

Finally, the report prepared by Sinke and Odegard (2021) provided insight into the environmental consequences that a commercial-scale production plant would have [37]. Data was collected among more than 15 companies that are active in the cultured meat sector and its supply chain. The functional unit was 1 kg of high-protein product, including cultured meat, conventional meat or a plant-based meat alternative eaten for its high protein content. The baseline scenario was modelled as hypothetical production in 2030, with several changes in the scale of production and electricity mix considered. Two electricity mixes were adopted: a conventional mix scenario (based on a global average of stated policy scenario for 2030) and natural gas for heating, and a sustainable mix scenario, using renewable energy (onshore wind turbines and solar PV panels) and geothermal energy for heat. Cultured meat was compared to conventional products including beef meat (beef cattle), beef meat (dairy cattle), pork meat, chicken meat, tofu and meatless (wheat-based) [37]. For the purposes of having a fair comparison and avoiding giving cultured meat advantages, changes to the conventional processes of these products were included to present an ambitious benchmark. A sensitivity analysis was carried out wherein the variables changed included production run time, maximum cell density, cell volume and efficiency of the medium use. Several impact categories from the ReCiPe World H/A method were assessed, such as global warming, land use and water consumption, among others. Results indicated that, in their baseline scenario, the global warming, expressed as the carbon footprint, ranged from 2.5 kg CO_2_-eq/kg product using sustainable energy sources to 13.5 kg CO_2_-eq/kg product using conventional energy. In the sensitivity analysis, their best-case scenario was the high-efficiency use of the medium combined with a sustainable energy mix. This resulted in a carbon footprint of 2.1 kg CO_2_-eq/kg product. The worst-case scenario is represented by a low efficiency of medium usage combined with a conventional energy mix. Here, the carbon footprint rose to 22.6 kg CO_2_-eq/kg product. In terms of energy consumption for the baseline scenario, the results ranged from 147 MJ/kg product when using sustainable sources of energy up to 264 MJ/kg product using conventional energy.

As expected, the differences in inventories and operational parameters are reflected in the results obtained by each study. Figure 1 depicts the variations between the resulting global warming of Mattick et al. (2015), Tuomisto et al. (2014) and Tuomisto and Teixeira de Mattos (2011) [26,28,35]. Energy use has not been included, since no record of the methodology used to calculate it was found in Tuomisto and Teixeira de Mattos (2011) and Tuomisto et al. (2014) [26,35]. Smetana et al. (2015) has not been included in the following analysis since they took a different approach in terms of system boundaries and LCA methodology [34].

The variation in the results, i.e., Mattick et al. (2015) obtaining around three times the global warming and energy use than other studies, can be partially explained by the inclusion of basal media production and the cleaning of the equipment [28]. Moreover, the feedstock and basal media formulations used in the study were based on peer-reviewed studies that proved their effectiveness to grow cells; however, empirical data would still be needed to prove if the use of only cyanobacteria hydrolysate is nutritionally enough for large-scale cell cultures [28]. Mattick et al. (2015) used the CML 2001 V 2.05 (100a) method to calculate global warming, while Tuomisto and Teixeira de Mattos (2011) and Tuomisto et al. (2014) used the IPCC method for 100a as well [26,28,35]. Even though this could represent some variation of the results, Mattick et al. (2015) calculated this value with both methods and indicated that the variation was only 0.5% [28]. In Sinke and Odegard (2021), the variation in values compared to the other studies is mainly because of the most robust, detailed, and complete life-cycle inventory [37]. Smetana et al. (2015) has a significantly higher amount of total environmental impacts compared to the other studies under analysis, which can be explained partly by the addition of extra steps to have a cradle-to-plate approach [34]. In terms of land use, Mattick et al. (2015) obtained higher results than the other authors; however, different methodologies to calculate this value were used by the authors, and, therefore, no comparison is possible [28]. Nevertheless, the difference of land use values compared to Tuomisto and Teixeira de Mattos (2011) can be explained by the use of different feedstock, including the addition of soy hydrolysate [26,28].

The differences in the life-cycle inventories between the studies make a comparison hard to achieve. Furthermore, the complexity of the data regarding cultured meat makes it a challenge to increase the accuracy of the environmental studies. To have detailed and precise LCA of cultured meat, it is necessary to consider all the processes and products involved in the core activities of cell growth, such as the ones provided Table 2. Taking into account other steps such as alternatives for waste treatment, sourcing of the materials and their supply chains will be of great importance when creating an environmental profile of cultured meat.

## 4. Discussion

### 4.1. Bridging the Gaps: Production Process Proposal for Future Assessments

**Figure 2:**
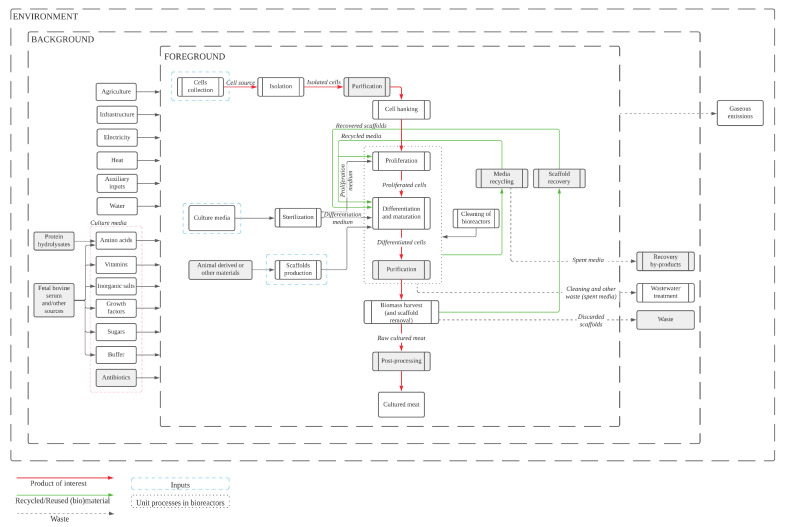
Process scheme proposal for future life-cycle assessments of cultured meat. Foreground system includes the core processes in the production of cultured meat, which will be quantified by the user. Background processes can be documented based on existing market data. Grey boxes indicate when the process can be excluded or when more than one option is possible. Boxes with single borders represent materials and biomaterials, while boxes with double borders represent unit processes.

Getting reliable data to assess the production of cultured meat is challenging at any scale, and therefore understanding the process and system boundaries is key. A proposal for a production process of cultured meat is presented in Figure 2. This process was based on information found in literature, as well as direct communication with experts in the field. This proposal provides a complete production process that can be included in future LCAs, with the addition of key steps compared to previous literature. The modelling of all the ingredients of the culture media, such as growth factors and vitamins, is one of the main differences to other studies. Cell collection, isolation and purification have not been included in previous system boundaries but do need to be accounted for. Furthermore, media processing, scaffold recovery and wastewater treatment are also key steps that have not been accounted for in the studied environmental assessments.

The foreground system contains processes that are specific to it and cannot be replaced by, e.g., market average supply data. As shown in Figure 1, key processes such as cell production (including collection, isolation, purification and storage) and scaffold production are all part of the foreground system, as well as the cell growth processes (and post-processes), medium recycling and scaffold recovery. On the other hand, the background system includes processes that can be appropriately represented by average, generic or equivalent market data [39], which in this case include the media ingredients’ production, agriculture, infrastructure, electricity, heat supply, auxiliary inputs, and water.

The cells’ collection can be achieved through a biopsy directly taken from the animal (e.g., muscle) to obtain adult stem cells with limited differentiation potential or in vitro fertilisation to produce embryonic stem cells with an unlimited capacity to differentiate into other cells. If the cells are not optimally isolated at the isolation step, an extra purifying process can be added to the system. Cells should be then stored at a cell bank, before entering the core growth process, which is divided in proliferation and differentiation [37]. A purification phase can be included in the process, which is mostly the case for embryonic stem cells or induced pluripotent stem cells, since the differentiation efficiency is lower than for other types. After being harvested, a post-processing stage has been added into the system in case the addition of extra components (e.g., vitamin B12) is needed [40]. Culture media are most often composed of amino acids, vitamins, inorganic salts, growth factors, sugars, a buffering system [41] and, in some cases, antibiotics. If preferred, all these ingredients can be obtained in commercially sold basal media formulations and supplemented with growth factors. In case all the ingredients are added independently, it is important to analyse their supply chains and methods of productions. For instance, some amino acids are commonly obtained from different sources [42], a common one being protein hydrolysates, which if used, would have to be considered in the system boundaries. Scaffolds are materials that will be key in the biological process happening in the bioreactors, as they serve as a support material for the cells to expand and differentiate [24]. Their production and potential recovery should be included in environmental assessments. The cleaning of the bioreactor is also considered as the main input, given the fact that they will have to go through constant cleaning processes after each production run [37].

As outputs, a media-processing step (e.g., recycling) has been included in the modelling. This would help mitigate the environmental impacts related to production. Where media recycling is not possible, potential by-products should be recovered (e.g., alanine, ammonia, lactate) to lower the environmental impacts [33], and finally, non-recycled or non-recyclable media should be treated as wastewater, along with the cleaning waste. Scaffolds are generally edible or degraded in the final product but if this is not the case, they should be removed at the harvesting step. The recovery of the scaffolds should be added where possible and otherwise treated as waste. Some microcarriers, such as washed cytodex-3, have been considered as reusable for mammalian cell cultures, as the rate of attachment, growth and metabolism of the cells on reused carriers were not affected compared to that of new ones [43]. Microcarrier beads could also be recoated before being reused [44]. Even though the recycling of these materials is normally not included in practice, due to the lack of economic or environmental needs, in food applications this could mean cost- and resource-efficiency in cell productions [44].

Not only the technical challenges of creating a process like the one described arise, but also gathering reliable data to generate a robust and complete life-cycle inventory to assess its environmental impact will have its difficulties. For this, several pathways can be followed, such as investigating the existing LCI databases, getting primary data from relevant actors in the industry or making assumptions based on literature.

### 4.2. The Challenge of Life-Cycle Inventories: Supply Chain Analysis

#### 4.2.1. Stem Cells: Collection, Isolation, Purification and Storage

Stem cell production is included in the foreground of the LCA model. The process starts with cell lines that can be directly obtained by doing a biopsy in a living animal [45] or created by genetically modifying them after taking cells from the source [46]. To produce cultured meat, several cell types have been considered and their suitability will depend on the capacity they show to self-renew and differentiate in a defined medium [24].

**Figure 3:**
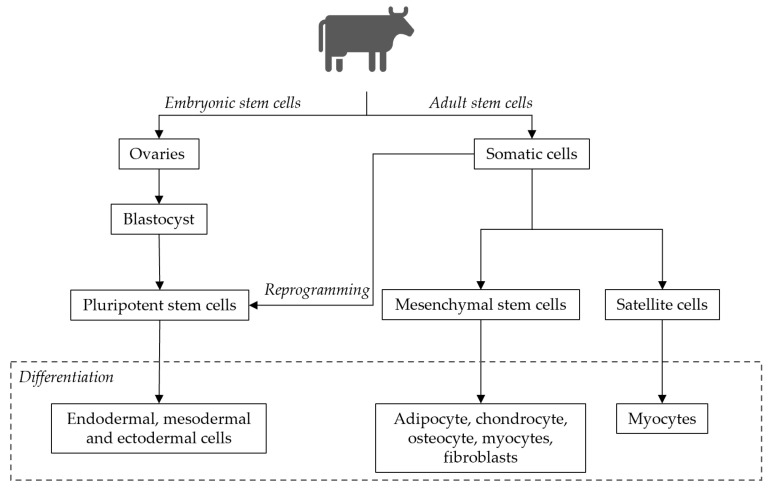
Classification of embryonic and adult stem cells from bovines. The reprogramming of cells refers to how induced pluripotent stem cells are obtained (original illustration based on [47]).

There are two categories of stem cells: embryonic stem cells (ESCs) and adult stem cells [47], as depicted in Figure 3. ESCs are derived from the inner cell of a blastocyst [48] and are pluripotent, meaning they have unlimited self-renewal and the potential for differentiation into cells corresponding to the three somatic germ layers that form an organism: mesoderm, ectoderm and endoderm [48]. To date, it has been a challenge in tissue engineering to establish cell lines from certain species, for example, bovines. However, under specific conditions, bovine ESCs (bESC) lines were efficiently established by Bogliotti et al. (2017), which opened the possibilities of producing cattle meat in vitro [49]. Adult stem cells are multipotent cells, meaning they can only differentiate into specific cell types correspondent to the germ layer from which they originated [47]. These cells can be directly isolated from several adult tissues. Mesenchymal stem cells (MSCs) are adult stem cells capable of differentiating into fibroblasts, adipocytes, osteoblasts, chondrocytes or myocytes [50], as seen in Figure 3. Because MSCs are mainly responsible for tissue repair, these cells are present in many, if not all, tissues [51]. Due to their abundance and ability to differentiate, MSCs have been considered a promising cell option for cultured meat production [52]. A common type of cell used in ongoing research to produce cultured meat are muscle stem cells, also referred to as satellite cells [53]. They are found in adult skeletal muscle, are easy to collect and do not require many inputs to differentiate into myotubes [24]. Furthermore, bovine satellite cells were used for the introduction of the first-ever cultured meat prototype [45]. To have a production of cultured meat on an industrial scale using satellite cell culture protocols, optimisation is still needed to increase their proliferation capacity [24]. However, Verbruggen et al. (2017) assessed their growth in a bioreactor and concluded that bovine satellite cells can actually be cultured in microcarriers [54]. Induced pluripotent stem cells (iPSCs) are adult cells isolated from somatic tissue that were reprogrammed into a pluripotent state [24] and therefore have infinite self-renewal potential. These cells can be engineered to achieve mass cell proliferation and therefore can potentially lower the cost of the process [52]. For these reasons, they have been considered to produce cultured meat. However, besides showing low yields [52], generating safe iPSC without genomic modifications remains a challenge [55]. This could in turn generate some issues related to consumer acceptance and regulatory procedures [52].

Regarding the collection of cells in the studied LCAs, Tuomisto and Teixeira de Mattos (2011) indicated that they did not consider the production of the embryonic stem cells used because one of these cells can produce more than 1000 kg of cultured meat and therefore is negligible [26]. This is not changed in Tuomisto et al. (2014) [35]. Smetana et al. (2015) mostly rely on the data provided in Tuomisto and Teixeira de Mattos (2011), and therefore the cell collection step was not included either [34]. The cultured meat production model presented in Mattick et al. (2015) started in the proliferation phase of the muscle cell cultivation, not considering the cell collection step [28]. To include this in future assessments, allocation procedures need to be considered. When allocating the environmental impacts related to agro-food systems, the main challenge is the multi-functionality of agriculture [56]. When there is a multiple-output situation, the production systems need to be allocated to these various outputs [57]. In the case of cattle, by-products represent around 44% of their liveweight [58]. Dairy cattle systems are multifunctional [59] and most commonly the main other output besides milk is meat from culled cows and surplus calves [60]. Beef cattle also have several outputs, including edible products (e.g., meat, blood, head, etc.) and non-edible ones (e.g., skin, tallow, manure, etc.).

A common method for cell isolation is enzymatic tissue digestion, in which the tissue is dissociated, and cells are harvested, through the use of enzymes, for instance collagenase and proteinase K [61]. A procedure for the isolation of satellite cells using this technique with the purpose of producing cultured meat was established by Lee et al. (2021) [62]. When considering larger-scale systems, the advantages of this technique are that it can be a low cost and automated process [61]. Another isolation process is the explant method. Its advantages for the isolation of human MSCs were reported by Hendijani (2017) [63]. When isolating cells with the explant method, enzymes are not used. In turn, the tissue is cut into small pieces and placed in dishes or flasks, where cells will start migrating out of the tissue into the culture surface [63]. When the objective is a large-scale setting, the author indicates that the latter method can be more cost-effective, given the lack of enzyme use. The purification of the isolated cells is not always necessary, and different protocols can be followed, for example, the purification of bovine satellite cells based on marker expression, as explained by Ding et al. (2018) [64]. A standard procedure to store cell lines is cryopreservation, which starts by cooling the samples for a short period of time and then and keeping them at very low temperatures, with e.g., liquid nitrogen (−196 °C) [65]. Stem cells encounter some issues when they are directly cooled down or frozen for long, since the formation of crystals, osmotic shock or membrane damage will kill them [65]. A hydrophilic cryoprotectant is regularly used to avoid intracellular ice crystal formation, such as glycerol or dimethyl sulfoxide (DMSO), the latter appearing to be more effective for this purpose [66].

None of the LCAs studied included these steps in their system boundaries. On the other hand, Sinke and Odegard (2021) start their production process at the banking stage, wherein vials with inoculum of cell lines are stored [37]. However, the contribution of the energy used for storage in the total carbon footprint of their environmental assessment is lower than 1%. Given this, it could be argued that the total contribution of processes such as collection, isolation, purification and banking to the environmental impacts of cultured meat production can be negligible. Nevertheless, to environmentally profile a production process as accurately as possible, these steps should be taken into consideration, especially when modelling large-scale systems.

#### 4.2.2. Scaffolds

Scaffolds are porous 3D structures [67] that play a significant role in the cell growth process, as they are a support network to which the cells can adhere to. These materials should be modelled in the foreground system. They allow the constant flow of nutrients and oxygen that help maintaining key metabolic functions of the cells [24]. The type of material chosen as scaffold will not only influence the cost of the process, but also the bioreactor’s efficiency, downstream processes, bioreactor’s fluid dynamics and mass transfer [68]. These parameters can affect the total environmental footprint of the process. Moreover, their design is of also great importance, as the surface area to volume ratio will determine the bioreactor size [68]. As previously mentioned, the scaffold can be edible, biodegradable or removed at the end of the process. Allan et al. (2019) indicated that one of the advantages of having edible biomaterials is that they can benefit the product’s texture, which can be useful at least until the co-culture of, for example fat cells, can be optimally achieved [68]. On the other hand, if the scaffold needs to be removed, dissociation techniques using animal-derived (e.g., trypsin) or non-animal-derived (recombinant enzyme TrypLE) enzymes can be used. Other techniques include mechanical dissociation or the use of an enzyme-free dissociation buffer such as Versene [68]. Physical, chemical and biological considerations for the biomaterials that can be used in agricultural applications are documented by several authors [24,67,69] and include biocompatibility, biodegradability, source, taste, nutrition, pore size, strength, architecture and manufacturing technologies, among others.

Animal-derived biomaterials for the scaffolds used in the production of cultured meat have been predominantly used, such as collagen and gelatine. Cultured meat’s main goal, which envisions environmental impact reduction and animal welfare, does not align with the use of animal-derived scaffolds, which is why plant-based alternatives are being considered for this purpose [67]. Materials derived from traditional livestock animals, like the above mentioned ones, cannot be replicated and would still involve the production of livestock [24], which is what is intended to avoid. Several types of biomaterials have been considered to use as scaffolds to produce cultured meat. They can be generally classified as natural polymers, including animal-derived and plant-derived, as well as synthetic materials. A summary of these, including the benefits and shortcomings related to each material, can be found in Seah et al. (2021) [67]. Some examples found include salmon gelatine, glycerol, chitin and more.

The reason behind the success of animal materials it is that they contain the characteristics of an extracellular matrix. Besides collagen, another one that has been used for cultured meat is gelatine, a meat component that was recently used to develop meat-like products when culturing bovine aortic muscle cells and rabbit skeletal muscle [67]. Fibrin is a naturally occurring protein that has been utilized in scaffolds as it optimises the vascularisation of bio-artificial muscles. Hyaluronic acid is known for wound healing and has also been widely employed for tissue engineering applications. Chitosan, another animal-derived product, has been already successfully used in skeletal muscles tissue engineering [67].

Polysaccharides are presented as an animal-free alternative to produce scaffolds. Bio-compatible and non-toxic polysaccharides like cellulose have already been used in tissue engineering applications [67]. Starch, another material sourced from plants, is also considered as an option for scaffolding material, though it is usually combined with synthetic polymers. Other polysaccharides options for scaffolds in cellular agriculture include cellulose and its derivatives, chitin, alginate and algarose [24].

Plant-based proteins present great advantages. They are widely available, are low cost and can be processed into several products used in medical applications [67]. When produced through recombinant techniques, protein-based materials such as fibrin, collagen, gelatine, keratin or silk can also be of interest as scaffolds in cultured meat production [24]. Other materials have been explored, such as soy-protein. This is a highly biocompatible and edible material investigated by Ben-Arye et al. (2020), where it was demonstrated that using textured soy protein can support cell attachment and proliferation, therefore being useful to create 3D engineered bovine muscle tissue [70].

Regarding synthetic polymers, the main advantage is the batch-to-batch reproducibility [67]. Examples of these are polylactic/polyglycol acids, polycaprolactone, polyethylene glycol and polyvinyl alcohol [24]. However, these materials need to be either edible or degradable without the use of toxic compounds. Even though some synthetic polymers are regarded as less biocompatible than natural polymers, a hybrid between both could be considered for application in cultured meat production [67]. There are other materials of interest that could fit the purpose. As expressed by Post et al. (2020), complex composites that can be sourced from plants or microorganisms include lignin, decellularized leaves and fungal mycelia, among others [24].

#### 4.2.3. Culture Media Ingredients

Culture media composition is a key parameter that will define the characteristics of the final cultured meat product [24] and maintain cells ex vivo [71]. The ingredients that compose it are part of the background system of the modelled LCA. Minimal essential medium (MEM) is a synthetic medium used to maintain cells in tissue culture that contains vitamins, amino acids, salts and glucose [72]. Many variations of this medium have been created, such as the commonly used basal media for mammalian cell cultures, Dulbecco’s modified Eagle’s medium (DMEM) [73].

To keep cells alive for a limited period of time, basal media formulations are usually sufficient, but for them to proliferate over longer periods, a variety of animal serums has historically been used [41], which presents a challenge for the sustainability of the system [24]. Foetal bovine serum (FBS) is the standard supplementation used for mammalian cell culture, as it is assumed to be the main source of growth factors. It is a complex mixture of components, such as vitamins, growth factors, trace elements, proteins and more, that are essential for the cells [73]. However, the use of this product poses several issues. First, it runs over the idea of not using or using fewer animals [24]. FBS is taken from the foetus of a slaughtered pregnant cow [74], which can in turn cause unnecessary pain to the unborn calf [73]. Animal-derived products not only introduce risk contaminations [24] (e.g., bovine spongiform encephalopathy) but also vary greatly from batch to batch. Since FBS contains around 200 to 400 different proteins and thousands of small-molecule metabolites in concentrations that are unknown [24], replacing it with chemically defined products can result in high costs and significant environmental burdens. The recyclability or recovery of the culture media will be key to determine the environmental profile of cultured meat. As indicated by Moritz et al. (2015), the recycling of culture media is in fact possible [44]. Ingredients that are used at high rates in the cell growth process, such as glucose and glutamine, can be replenished for new media entering the system, and waste products, such as lactate and ammonia, can be removed. Furthermore, as indicated by the mentioned authors, growth factors and cytokines are produced by the cells and can stimulate further growth; hence, the reuse of part of the media should be considered beneficial. Operationally, media recycling could be achieved “on-line”, meaning that the cells could stay in the bioreactor while the media is being recycled and the unwanted waste-products are being discarded [44].

The inaccuracy of environmentally profiling culture media is unavoidable since there is poor information regarding the environmental impacts of the ingredients. Several LCI databases were checked (i.e., ecoinvent, Agri-footprint, GaBi, ELCD, Agribalyse, GFLI, World Food LCA) to determine if the components of basal media formulations were documented. None of the vitamins, growth factors or buffer agents used in common formulations were found in databases. Sugars, for which the production is well-established, were found in one or more LCI database. Around 42% of the amino acids were documented in one or more databases and approximately 70% of the inorganic salts. This is an indication of the complexity of accurately defining a medium composition and assessing it from an environmental point of view.

Tuomisto et al. (2014) indicated that one of the main uncertainties that consequently affected the results of the study was the culture medium composition [35]. Only the impacts of producing the main ingredients of the medium were included and have a major contribution on the environmental burdens of the process. Smetana et al. (2015) suggested that one of the reasons for the large difference in the environmental impacts between the meat substitutes under their analysis might be attributed to the high energy consumption by cyanobacteria medium growth for the culture [34]. Mattick et al. (2015) reported that the inclusion of the production of the media resulted in an energy consumption and GWP three times higher than the one calculated by Tuomisto and Teixeira de Mattos (2011) [26,28]. Furthermore, nearly all the energy use was only used to produce the media. Finally, Sinke and Odegard (2021) concluded that the main drivers of the environmental impacts related to cultured meat production were electricity use during the production phase and, again, the medium production [37]. More specifically, they indicated that producing albumin largely contributed to the whole environmental footprint when compared to other ingredients. In summary, the recycling of the media would reduce the natural resources use, energy use and the amount of wastewater [37].

Amino acids

Amino acids are involved in the production of proteins and other low-molecular-weight compounds, such as peptides and nucleotides, and can be classified as essential and non-essential [41]. All of the essential amino acids that are needed to culture cells are present in high concentrations in DMEM [73]. These include arginine, cysteine, glutamine, histidine, isoleucine, leucine, lysine, methionine, phenylalanine, threonine, tryptophan, tyrosine and valine. Non-essential amino acids can be found either in other nutrient-rich basal media formulations or alternatively added independently [41]. Amino acids can be produced through several processes. These include extraction from protein hydrolysates, chemical synthesis or enzymatic and microbial fermentation [42]. The benefits and drawbacks of each production process were documented by D’Este et al. (2018) [42].

Tuomisto and Teixeira de Mattos (2011) indicated that their source of nutrients for cell growth was cyanobacteria hydrolysate, as previously indicated [26]. No details were given in terms of specific amino acids present in the feed, and no supplementary amino acids were included in the study. The same principle was followed by Tuomisto et al. (2014), where the replacement of cyanobacteria-based media with plant-based media was assessed [35]. Yet again, no specific information about which amino acids are present in the media, and no supplementary sources were added into the feed. Mattick et al. (2015) assumed the use of a serum-free media supplemented with soy hydrolysates as feedstock. Two pathways to produce amino acids were included in the study, which were the soy hydrolysate production and glucose fermentation. For the latter, allocations procedures were done in terms of gross chemical (calorific) energy basis (GCE) [28]. As stated by Sinke and Odegard (2021), hydrolysate sources have a lower environmental impact per kg than other amino acids sources, such as microbial- or synthetic-based [37]. These sources should therefore be prioritised in the production of amino acids for cultured meat.

When including amino acids in the production of culture media for a life-cycle assessment, it is important to understand their supply chains, whether they are manufactured only for the purpose of cultured meat or, if they are considered by-products of other industries, which of their impacts will have to be partly allocated to cultured meat. Furthermore, certain processes such as extraction from protein-hydrolysates do not generate a great variety of amino acids [42] and therefore will always need a secondary source for cell cultures. A limited number of amino acids have been found in LCI databases, so, to understand the contribution of these ingredients to the environmental impact of the culture media, more should be modelled in the future.

Vitamins

Vitamins are classes of organic compounds usually used for specific intracellular functions [75]. In vivo, vitamins are typically processed in a complex sequence due to the presence of hostile environments, such as stomach acids. This is avoided when growing cells in vitro, and therefore vitamins are added as single chemical compounds that are directly absorbed by the cells [41]. In common media formulations, at least seven vitamins are considered essential for growth and proliferation: choline, folic acid, nicotinamide, pantothenate, pyridoxal, riboflavin and thiamine [73]. Vitamins that are excluded but necessary for the process can be added independently. Commonly, they can be produced through chemical synthesis or fermentation [76]. Each process has its own advantages. Chemical paths include chemical mutagenesis, the application of N^+^ ion beam, ultraviolet radiation or laser mutagenesis [77]. The chemical synthesis of vitamins requires high temperatures or pressurised reactors and the use of non-renewable chemicals or toxic solvents, all of which can result in significant pollution and hazardous waste [77]. On the other hand, fermentation methods, which include the construction and mutagenesis of the starting strain, genetic modification, synthetic biotechnology and others [77], have been described as a more environmentally friendly option [76]. It has also been stated that it poses a more economically sustainable alternative.

To include the production of vitamins in LCAs, it is important to consider which vitamins are being commercially produced within each method. As literature suggests, microbial methods are gaining attention due to the benefits their process poses for the environment [76], which in the case of cultured meat, should be prioritised.

Inorganic salts

Inorganic salts establish and maintain the osmolarity of the cells and the surrounding medium [41]. The originally formulated minimal essential medium is composed of six inorganic salts: calcium chloride, magnesium sulphate, potassium chloride, sodium bicarbonate, sodium chloride and sodium phosphate monobasic. Besides the mentioned ones, some media formulations include additional inorganic salts, which play important roles in cellular functions [28].

Their production, obtained by chemical synthesis, must be included in the production of the culture media when carrying out life-cycle assessments. From the analysed studies, only Mattick et al. (2015) included them in the life-cycle inventory; however, no highlight regarding their contribution to the total environmental impact of the media production is given [28]. Sinke and Odegard (2021) also documented the use of salts in their media composition; however, no attention was provided in terms of environmental impacts [37]. This could suggest that their contribution to the total environmental impacts related to the culture media is significantly low compared to other ingredients.

Sugars

Cells in culture need energy inputs. The most common sugar used for this purpose is glucose, or D-glucose, although other formulations might include a mix of glucose and pyruvate [41]. The production of glucose is associated with little waste production and high integration, in which 57% of the electricity and 59% of the heat are produced via a combined heat and power system [24]. Glucose is produced by the hydrolysis of raw materials, for example starch, naturally produced through the photosynthesis of plants, which in turn requires land and water [24].

Mattick et al. (2015) included the production of corn starch and glucose from wet corn starch in their calculations. Per kg of cultured meat produced in their study, glucose contributed 4.05 MJ of energy use and a global warming potential of 0.37 kg CO_2_-eq [28]. Even though these numbers are not significantly high compared to other ingredients, such as glutamine, to achieve an environmentally friendlier culture media, the ingredients needed in high concentrations, like glucose, should be sourced and dosed carefully [24]. This is especially important when considering the supply chain of glucose, in which the agricultural phase is responsible for the highest amounts of environmental impacts [78].

Growth factors

Growth factors, usually proteins or hormones, are essential to culture processes and the great reason why FBS is so successful as a growth supplement [41]. There is limited information regarding which growth factors are present in FBS, but it has been reported that typical ones include members of the insulin growth factor family, fibroblast growth factor family, transforming growth factor family and neuregulin family [79]. Growth factors activate signalling pathways, which in turn regulate the cellular activity, stimulating the proliferation and differentiation of the cells [24] and are therefore a key ingredient in culture media. Proteins needed in the process, such as albumin, are produced recombinantly. These proteins are manufactured inside host cells, commonly of a different species, and are called recombinant because their DNA has been recombined or engineered [80]. Given the high demand for growth factors from different industries, several expression systems, such as *Escherichia coli, Bacillus subtilis*, mammalian cell, baculovirus, silkworm and plant systems have been used to manufacture them [81].

Producing growth factors recombinantly has resulted in high costs and significant environmental impacts. No information is given about the production of growth factors and the resulting consequences on any of the LCAs published. However, Sinke and Odegard (2021) indicated that the production of growth factors and other recombinant proteins, such as albumin, contributed to the highest fraction of the total carbon footprint of the medium used per kg of cultured meat [37]. This is extremely important, especially since growth factors were added in amounts smaller than 1 g in all their scenarios. This means that for future LCAs, even a small change in the concentration of growth factors will result in meaningfully lower environmental impacts related to the media composition.

Buffering agents

Buffering systems are essential, as they are used to maintain the pH of the cells, which needs to be around 7.4 ± 0.4, at a constant level, even when composition changes could induce variations in the pH of the medium [41]. These are a mixture of a weak acid and its conjugate base or a weak base and its conjugate acid. Furthermore, in cell cultures, buffers usually consist of CO_2_-bicarbonate systems or agents such as HEPES ((4-(2-hydroxyethyl)-1-piperazineethanesulfonic acid) [41]. HEPES is widely used and is one of Good’s buffers, which are 20 buffering agents that fit the criteria to be used in biological and biochemical studies, defined by Norman Good and his research team [82].

In fact, Mattick et al. (2015) and Sinke and Odegard (2021) used it in their life-cycle assessments [28,37]. However, in terms of carbon footprint, the buffering agent is not highlighted as relevant. Nevertheless, it has been described as the main driver of the economic cost of commercial media formulations [83], so the amount used should be considered with caution.

Antibiotics

In addition to FBS, antibiotics use poses some issues when producing cultured meat. These have been commonly added to avoid contamination in the cell cultures, even though all the start-ups have claimed this problem does not exist anymore [30]. The biggest argument against the use of antibiotics in livestock is antibiotic resistance [30]. Even though they are used in a controlled environment, under strict surveillance and to prevent early contamination when producing cultured meat, the argument remains valid [30]. From an environmental perspective, there are several issues related to the use of antibiotics in general, whether it is as human medicine or in agricultural production processes. In low concentrations, traces of antibiotics have been detected in groundwater, seawater, surface water and most shockingly, in drinking water [84]. Besides having been identified in potable water, when absorbed in soils, antibiotics have shown not to be readily degraded [85].

Antibiotics are usually produced through fermentation. Several types of antibiotics have been isolated from microbes [86]. Other pathways to the manufacture of antibiotics include their synthetic production, wherein they are entirely produced in the laboratory, which due to the complexity of their molecules, is rarely the case. Semi-synthetic processes provide a halfway point between natural fermentation and synthetic pathways. These antibiotics are a combination of the fermentation and of laboratory work, wherein the active part of a natural antibiotic is slightly modified. Neither the production of antibiotics nor their use was included in the studied LCAs. Sinke and Odegard (2021) did not refer to any [37], which could suggest that companies and actors related to the supply chain for the production of cultured meat are, in fact, not using them. If included in future hypothetical modelling of cultured meat processes, they should be considered for the environmental assessment as well. However, it is recommended to find primary data, especially from companies producing cultured meat, to avoid the use of antibiotics for this purpose.

#### 4.2.4. Equipment and Infrastructure

Each phase of the cell growth process, i.e., proliferation and differentiation, takes place in bioreactors. Their goal is to provide a stable and controlled environment that is suitable for the management of mammalian cells [68]. To choose and design the best bioreactor configuration, several requirements should be considered. The final product, whether this is a processed or full cut meat, a dry protein powder or wet cell biomass [68], will influence upstream and downstream processes. The design is an iterative process and besides calculating mass and energy balances, heat supply, removal and energy savings, other upstream processes need to be included such as storage tanks, heat exchangers and means of maintaining the right conditions in the bioreactors [68]. The referenced authors provide a summary of the key areas to consider in the design of bioreactors to produce cultured meat, highlighting that the main challenge for the industry is cell expansion via proliferation at an industrial scale [68]. The bioreactor’s configuration and operation mode can be batch, fed batch or continuous. Any choice will impact the sizing and media requirements [68]. In large-scale settings, usually a fed-batch or a continuous supply of media is preferred. In biotech industries, agitated vessels are the most used, given that they provide a time-averaged homogeneous and mixed environment [68]. The stirred-tank bioreactor is the standard for mammalian cell culture, as it has advantages for scalability. However, it can give high shear stress to the cells given the mechanical agitation that is needed for mixing [24].

Tuomisto and Teixeira de Mattos (2011) assumed the use of stirred-tank bioreactors for cell cultivation. The manufacture was included in their calculations, and they assumed to be made of stainless steel [26]. As indicated by the authors, the production of 1 kg of stainless steel requires 30.6 MJ of energy and emits 3.38 kg CO_2_-eq. The lifetime of the bioreactors was assumed to be 20 years. The heating of the bioreactors was not included, since, as indicated by the authors, it is not required due to the heat produced by the cells themselves. One of the changes implemented in Tuomisto et al. (2014) is that the bioreactor is now assumed to be a hollow fibre bioreactor [35]. Unlike Tuomisto and Teixeira de Mattos (2011), this assessment did include the heating and pumping energy, balancing them against the heat generated by the cells [26]. The bioreactor was assumed to be made of 5 mm thick stainless steel, the lagging made of 25 mm thick glass wool and the hollow fibres made from polylactide. Mattick et al. (2015) modelled both the proliferation and differentiation phases in a stirred-tank bioreactor [28]. The manufacturing of it is not included, however, the cleaning and heating of it is. Sinke and Odegard (2021) modelled the use of stirred tank bioreactors, with three different volumes: 50 L, 2000 L and 10,000 L. The production, heating, cooling and cleaning of the bioreactors are included in their analysis [37]. The main difference with Mattick et al. (2015), is the inclusion of active cooling to the bioreactors to avoid overheating, which is reflected in the results, by it being the main driver for electricity use (91%) in large-scale proliferation [28].

Mattick et al. (2015) did include the facility production in their calculations in order to keep consistency when comparing it to the referenced livestock LCAs [28]. Their building size was assumed to be similar to the smaller footprint of a brewery. The other studies under analysis did not include facility production in their assessments.

In LCAs, the contribution of the infrastructure production to the total environmental impacts is often negligible, especially for energy-intensive processes [28] such as cultured meat. Equipment and infrastructure can be part of the background system. Its inclusion will play a more crucial role when modelling lab-scale processes but will, at the same time, add uncertainty for new technologies [33]. Therefore, when assuming the production of cultured meat will be at an industrial scale, including infrastructure can be useful, but is not essential.

#### 4.2.5. Energy Sources

The choice of energy sources in the background system, especially electricity, will be significantly reflected in the results of a life-cycle assessment. As presented in Section 3, the LCA published by CE Delft considered two different energy mixes to assess the environmental impacts of cultured meat [37]. Results showed that the environmental impacts (ReCiPe single score, per kg product) of cultured meat when using conventional energy sources (354 mPt/kg cultured meat) more than doubled the ones calculated when switching to sustainable sources of energy (130 mPt/kg cultured meat). The total carbon footprint of cultured meat using conventional energy was more than five times higher than the one using sustainable energy sources. Therefore, choosing the right energy source will be key in future environmental assessments.

Sinke and Odegard (2021) indicate that, when modelling the future of cultured meat under a sustainable energy scenario, it is important to also consider certain changes in the production processes of conventional meat products, to avoid giving an advantage to cultured meat and allow a fair comparison between products [37]. This means that when the goal is to compare the environmental performance of cultured meat to another type of protein, the energy used in both models should be similar. Different electricity and heat sources can also be included in a sensitivity analysis [33].

### 4.3. Challenges from a Life Cycle Perspective

#### 4.3.1. Goal and Scope

System boundaries

An attributional LCA aims to estimate the amount of environmental burden associated with the product or process under study. In a consequential LCA, the consequences of producing and using the studied product or process and how they affect the global environmental burdens are estimated. All the studies under analysis performed attributional LCAs. Since the goal of an attributional LCA is to understand the environmental impact of a product’s life cycle, following this method is understandably the right choice for future LCAs. Cultured meat is a new product, not massively offered in the market, and therefore, before being concerned about modelling alternative courses of action as it is with a consequential LCA, the environmental impact of the product itself should be investigated first.

The scope of an LCA is determined by its system boundaries. Typically taken system boundaries are cradle-to-grave or cradle-to-gate. The latter considers the life cycle of a product until it reaches the factory gate, while the first includes use and disposal phases [87]. All the studies under analysis took a cradle-to-gate modelling approach, except for Smetana et al. (2015), who took a step further and modelled until the product reached the final consumers. Transportation of the product to a supermarket, cooling on a cooling counter, other product additions (vegetable oil, salt), transportation to the consumer and frying on an electric stove were included in their calculations [34]. Due to the high uncertainty of the already existing LCAs of cultured meat, processes beyond the factory gate would increase the inaccuracy of the results, as they would be entirely based on assumptions and literature and not so much on primary data.

Functional unit

The functional unit varied between the studies considered. Tuomisto and Teixeira de Mattos (2011) considered 1000 kg of cultured meat with a dry matter content of 30% and protein content of 19% of the mass [26]. However, the comparison of cultured meat to conventionally produced European meat, such as beef, pork, and poultry, was done on a weight basis, not considering either the protein content or the dry matter content of the product. For the beef produced in Ireland, Tuomisto and Teixeira de Mattos (2011) used the results of an LCA carried out by Casey and Holden (2006), which does not consider the protein content of the meat, but only the live weight of the cow [26,88]. Nutritional values of meat are not considered either when comparing it to the pork and beef production systems in Sweden, provided by Kumm (2002) [89]. Again, when the impacts of cultured meat were compared to the ones reported in the UK by Williams et al. (2006) for poultry, pork, beef and lamb, the nutritional values of the meat were not considered [38].

Tuomisto et al. (2014) allocated all the impacts towards the same functional unit as Tuomisto and Teixeira de Mattos (2011) [26,35]. Furthermore, the energy use for beef, pork, sheep and poultry was again compared to the ones documented by Williams et al. (2006) [38], in terms of edible meat. GHG emissions and land use were on the other hand compared per unit of protein, based on the data provided by Nijdam et al. (2012) [90]. When comparing the results of cultured meat to conventional meat sources, Smetana et al. (2015) did it based on the calorific energy content (3.75 MJ, approximately 896 kcal) of a ready-for-consumption product, which resulted in a functional unit of 0.3 kg of cultured meat [34]. An alternative functional unit was included in the sensitivity analysis, based on the digestible bulk protein content, which was determined to be 0.3 kg of the protein product (dry weight). This resulted in a cultured meat product with 26% protein content, which is reached with 1.25 kg of product [34]. Mattick et al. (2015) compared the environmental impacts that resulted from their assessment to beef, pork and poultry [28]. The comparison of cultured meat to those three other meat products was based on the work provided by three studies (see Mattick et al. (2015) for more information). However, these were all done based on the mass of the edible portion of the live weight of the animal, not considering the nutritional values that each provides.

Comparisons of food products based on the weight of the meat can lead to false conclusions and is often not sufficient [33]. When relating environmental impacts to the mass of a product, the real functions that food offers are not taken in consideration. These can include nutrient supply, pleasure, satiety, among others [91]. However, basing the calculations only on protein content can also be limiting, as it dismisses the fact that different products have different protein digestibility [91]. Protein is not the only important nutrient that meat provides to humans. Minerals, proteins, fat, fatty acids, cholesterol, salt and vitamins, such as B12, are important components provided by meat as well [92]. Choosing the right functional unit for food products will have a significant impact on the results. While some studies have shown that diets with more meat, vegetables and fruit have been associated with higher GHGs, others have stated that replacing red meat with equicaloric amounts of vegetables and fruit, led in fact to higher GHGs [93].

For future assessments of cultured meat, to allow a fair comparison to other meat or meat-like products, a more detailed nutrient-based functional unit should be applied, for example, based on B12 content, protein content or fat content. This has been advocated by different authors, such as Schau and Fet (2007), who proposed the use of a quality corrected functional unit (QCFU), that considers the quantity, fat content, protein content and carbohydrate content of a food product [94]. Heller et al. (2013) reviewed more than 30 studies that used an LCA framework to assess diets and meals [95]. They conclude and map functional unit choices for different foods, using a more comprehensive nutritional basis to link the environmental impacts and their nutritional value.

#### 4.3.2. Data Collection

Getting reliable data for cultured meat has proven to be complex. Empirical data is often highly confidential, and therefore, the existing studies have mainly had to rely on assumptions and literature. Tuomisto and Teixeira de Mattos (2011), as well as Tuomisto et al. (2014), used stem cells for the production of cultured meat, which at the time they were written, lacked any type of industrial data [33]. On the other hand, Mattick et al. (2015) based their life-cycle inventory on CHO cell cultivation, since the metabolic requirements for muscle cell cultivation were not found [28]. These differences already determine the operational parameters needed in further processes, such as the material and energy flows [33]. The culture media used between these studies varied greatly; nevertheless, Mattick et al. (2015) made use of one similar to commercial basal media for the cultivation of mammalian cells, combined with other components [28]. However, it was assumed that the production of half of the amino acids for the basal media followed a process similar to lysine and the other half to threonine. Even though Tuomisto and Teixeira de Mattos (2011) used existing lab-scale data regarding the cyanobacteria biomass flows, it is not clear whether this media is sufficient to produce cultured meat at a large-scale facility [26]. The production of certain key components, e.g., growth factors, was not added in any of the mentioned publications.

Other differences that can be highlighted are the type of bioreactors, scaffolds, feedstock and cell source used, which, as mentioned in Section 3, vary in most of the studies. As reported by Scharf et al. (2019), since there is no standard commercial bioreactor for the production of cultured meat, the design and energy needed for them will remain uncertain [33]. In addition, certain parameters, such as agitation, aeration, pumping and others, are based on calculations that depend on the properties of the equipment [33]. Other key parameters included in the life-cycle inventories that varied throughout the studies included initial cell densities, maximum cell densities and times per batch, among others. Given this, it is important that the gaps, especially the ones related to core processes, are filled with methodologically consistent data to increase the quality of the life-cycle inventories. Preferably, primary data, in which the sources are the producers of goods and operators of processes and services [39], can be prioritised when modelling the cultured meat process. Primary and measured data, whether it is from a lab-scale or commercial-scale source, will increase the reliability of the results. Considering similar processes to the ones intended to model is also suitable for non-existing technologies. Even though it is expected that LCAs carried out in the first stages of development of new technology have more environmental impacts than industrial-scale processes [33] and therefore should not be considered as final results, they nonetheless serve as a contribution to the understanding of the technology under study [96].

Including experts’ judgement and modelling can be of use to fill the gaps of the data inventory when assessing the upscaling of a non-existent technology. Gavankar et al. (2015) introduced the concepts of technology readiness levels (TRL) and manufacturing readiness levels (MRL) and studied what their roles are in LCAs [97]. They conclude that the outcomes of life-cycle assessments of emerging technologies should be analysed in relation to their scales. This is because lower-scale models most likely cannot be scaled up linearly to higher levels [98]. In the case of cultured meat, the use of learning curves is not directly applicable since there is no previous market-based experience with this technology. However, van der Giesen et al. (2020) indicate that, when this is the case, similar technologies can in fact provide an insight into how the development will be in the future, combined with regular laws of physics and critical expert input [98]. The challenge of assessing prospective models can also be solved by combining learning curve insights with experts that can provide knowledge and experience when it comes to upscaling processes [98]. The most feasible solution for the challenge of modelling future and non-existent technologies is to have structured discussions with experts, with the objective of gathering information and conducting different scenarios based on it [98].

#### 4.3.3. Multifunctionality

The decision hierarchy on which type of allocation to use will depend on how complex the data is. To avoid allocation, the product environmental footprint (PEF) recommends a subdivision or system expansion. Subdivision is the disaggregation of multifunctional processes or facilities, isolating the input flows associated with each process of the facility. System expansion happens when the system is expanded by including additional functions related to the co-products of the process. Nonetheless, this is often not possible due to the complexity of the system expansion, especially in agri-food products, wherein the existing alternatives usually also come from multifunctional systems [56]. When it comes to the meat supply chain, many products and co-products are generated, with many uses and actors involved [56]. Furthermore, when deciding on allocation procedure, the sector could not reach a consensus on which proved to be more suitable.

There are other methods to allocate: economic allocation and physical (e.g., mass) allocation. Economic allocation is when the environmental impacts of a certain process are allocated to each output based on their relative economic value [57]. Physical allocation takes place when the impacts are allocated to each output based on their physical properties, such as mass, dry mass, volume, energy content and so on. These two types of allocation are the most widely used for food product studies due to ease of data collection [99]. If some of the ingredients of cultured meat are agricultural products, such as the culture media, animal-derived products and others, the multifunctionality of the original source must be considered. In this regard, it is important to understand that the allocation for agri-food products, especially livestock, remains a challenge. Even though other types of allocations have been suggested for these products [100], Wilfart et al. (2021) indicate there was “no consensus reached among partners in the context of the European Product Environmental Footprint of meat products” [56]. Throughout the studies, different allocations methods were used for other processes or materials. For example, Tuomisto and Teixeira de Mattos (2011) did not allocate the potential energy generated at the anaerobic digester by the cyanobacteria not suitable for muscle cell cultivation, which in turn, could help lower the impacts allocated to cultured meat [26]. Thus, accounting and allocating for any valuable side-stream, the recovery of biomaterials and the recycling of media streams will be key when determining the environmental profile of cultured meat. To understand the impact of which type of allocation was chosen, it is recommended to study them in a sensitivity analysis, to obtain a fair conclusion.

## 5. Conclusions and Recommendations

Several limitations were encountered when carrying out this investigation. As explained, published life-cycle assessments of cultured meat are scarce, and most of their life-cycle inventories have high uncertainty. It has been concluded that cultured meat has the potential to overcome several issues related to, especially, environmental concerns. Consequently, its introduction to the market will benefit animal welfare as well. The future of the environmental assessments of cultured meat will depend on how much reliable data is developed in the following years. Ideally, primary data, obtained in collaboration with the relevant actors of this sector, can facilitate the creation of an accurate life-cycle inventory. Even though this work is based only on the environmental impacts of cultured meat, other limitations that can be foreseen include consumer acceptance and the social and economic consequences that this novelty will bring.

No complete nor consistent life-cycle assessment of cultured meat has been conducted, owing to the lack of information related to the processes and materials. Nevertheless, it has been concluded that there are a few key steps in the production system of cultured meat that are essential to considering the total environmental impacts. Some of these processes include media production, with a special focus on growth factor production, scaffold production, after-use media processing, the recovery of by-products and others. Cultured meat has the potential to be an environmentally friendly product compared to conventional meat-based products. However, switching to more sustainable sources of energy will be key to determine this outcome. On the other hand, the availability of these processes and the biomaterials involved is extremely limited in the LCI databases. The information gaps emerged as a major barrier to an accurate and complete life-cycle assessment of cultured meat.

This study has paved a path for future environmental assessments of cultured meat. In essence, LCA practitioners can use this framework as a starting point for future research, as it has also been indicated which processes and materials are key to consider when environmentally profiling this emerging technology. While it is acknowledged that this is a preliminary proposal and many processes may vary in time, it serves as a basis for upcoming assessments. Future research should include the collection of data as accurate as possible, ideally in collaboration with relevant actors in the industry to model the ingredients and processes missing in the LCI databases. These steps will be key in bridging the gap of knowledge that has been highlighted in this article. Green supply chain management, which represents the integration of environmental thinking applied to supply chain management [101], can be combined with tools such as LCA to increase the environmental responsibility of food production systems in the future.

## Figures and Tables

**Table 1:** Life-cycle assessment and operational parameters of the analysed studies.

	Tuomisto and Teixeira de Mattos (2011)	Tuomisto et al. (2014)	Smetana et al. (2015)	Mattick et al. (2015)
Functional Unit	1000 kg cultured meat ^1^	1000 kg cultured meat ^2^	Satisfaction of a consumer with 1 kg protein-enriched product ready for consumption ^3^.	1 kg of Chinese hamster ovary (CHO) biomass ^4^
System boundaries	Cradle-to-gate	Cradle-to-gate	Cradle-to-plate	Cradle-to-gate
LCI modelling principle	Attributional	Attributional	Attributional	Attributional
LCIA method	IPCC 2006	IPCC 2006	ReCiPe V1.08 and IMPACT 2002+	Cumulative energy demand, ecological footprint and CML 2001
Cell type	Stem cells from animal embryo	Stem cells from animal embryo	Stem cells from animal embryo	CHO
Feed source	Cyanobacteria hydrolysate	Cyanobacteria hydrolysate, wheat, and corn	Cyanobacteria hydrolysate	Serum-free media supplemented with soy hydrolysate
Bioreactor type	Cylinder stirred tank	Hollow fibre	Cylinder stirred tank	Stirred tank
Production time	60 days	90 days	60 days	11 days

^1,2^: Dry matter (DM) of 30% and protein content of 19%. ^3^: Functional unit used for the main study. In the sensitivity analysis, the authors assess two other functional units. ^4^: Dry matter (DM) of 17% and protein content of 7%.

**Table 2:** Overview of materials and processes considered within or outside the scope of each study under analysis. Positive sign (+) indicates these processes or materials were included in their life-cycle inventories, while negative (−) sign indicates these were excluded.

	Tuomisto and Teixeira de Mattos (2011)	Tuomisto et al. (2014)	Smetana et al. (2015)	Mattick et al. (2015)
Cell collection	−	−	−	−
Growth factors production	−	−	−	−
Scaffold production	−	−	−	+
Bioreactor’s production	+	+	+	−
Cleaning bioreactor	−	−	−	+
Culture media recycling	−	−	−	−
Scaffold removal/recovery	−	−	−	−
Wastewater treatment	−	−	−	−

## References

[1] Steinfeld H., Gerber P., Wassenaar T., Castel V., Rosales M., de Haan C. (2006). Livestock’s Long Shadow: Environmental Issues and Options.

[2] Thornton P.K. (2010). Livestock production: Recent trends, future prospects. Philos. Trans. R. Soc. B Biol. Sci..

[3] Bouwman L., Goldewijk K.K., Van Der Hoek K.W., Beusen A.H., Van Vuuren D.P., Willems J., Rufino M.C., Stehfest E. (2013). Erratum: Exploring global changes in nitrogen and phosphorus cycles in agriculture induced by livestock production over the 1900–2050 period. Proc. Natl. Acad. Sci. USA.

[4] Aiking H. (2011). Future protein supply. Trends in Food Science and Technology.

[5] Hagmann D., Siegrist M., Hartmann C. (2019). Meat avoidance: Motives, alternative proteins and diet quality in a sample of Swiss consumers. Public Health Nutr..

[6] Tukker A., Goldbohm R., De Koning A., Verheijden M., Kleijn R., Wolf O., Pérez-Domínguez I., Rueda-Cantuche J. (2011). Environmental impacts of changes to healthier diets in Europe. Ecol. Econ..

[7] Errickson F., Kuruc K., McFadden J. (2021). Animal-based foods have high social and climate costs. Nat. Food.

[8] Ripple W.J., Smith P., Haberl H., Montzka S.A., McAlpine C., Boucher D.H. (2014). Ruminants, climate change and climate policy. Nat. Clim. Chang..

[9] Alvarez R.A., Pacala S.W., Winebrake J.J., Chameides W.L., Hamburg S.P. (2012). Greater focus needed on methane leakage from natural gas infrastructure. Proc. Natl. Acad. Sci. USA.

[10] Myhre G., Shindell D., Bréon F.-M., Collins W., Fuglestvedt J., Huang J., Koch D., Lamarque J.-F., Lee D., Mendoza B., Stocker T.F., Qin D., Plattner G.-K., Tignor M., Allen S.K., Boschung J., Nauels A., Xia Y., Bex V., Midgley P.M. (2013). Anthropogenic and Natural Radiative Forc-ing. Climate Change 2013: The Physical Science Basis. Contribution of Working Group I to the Fifth Assessment Report of the Intergovernmental Panel on Climate Change.

[11] IPCC Climate Change 2021: The Physical Science Basis. Contribution of Working Group I to the Sixth Assessment Report of the Integovernmental Panel on Climate Change 2021. https://www.ipcc.ch/report/ar6/wg1/#FullReport.

[12] Mekonnen M.M., Hoekstra A.Y. (2010). The Green, Blue and Gray Water Footprint of Farm Animals and Animal Products.

[13] De Backer C.J.S., Hudders L. (2015). Meat morals: Relationship between meat consumption consumer attitudes towards human and animal welfare and moral behavior. Meat Sci..

[14] Godfray H.C.J., Aveyard P., Garnett T., Hall J.W., Key T.J., Lorimer J., Pierrehumbert R.T., Scarborough P., Springmann M., Jebb S.A. (2018). Meat consumption, health, and the environment. Science.

[15] Alonso M.E., González-Montaña J.R., Lomillos J.M. (2020). Consumers’ Concerns and Perceptions of Farm Animal Welfare. Animals.

[16] Cornish A., Raubenheimer D., McGreevy P. (2016). What we know about the public’s level of concern for farm animal welfare in food production in developed countries. Animals.

[17] Broom D.M. (2010). Animal welfare: An aspect of care, sustainability, and food quality required by the public. J. Vet. Med. Educ..

[18] Craig W.J. (2009). Health effects of vegan diets. Am. J. Clin. Nutr..

[19] Dagevos H. (2014). Flexibility in the Frequency of Meat Consumption—Empirical Evidence from The Netherlands. EuroChoices.

[20] Derbyshire E.J. (2017). Flexitarian Diets and Health: A Review of the Evidence-Based Literature. Front. Nutr..

[21] Asgar M.A., Fazilah A., Huda N., Bhat R., Karim A.A. (2010). Nonmeat Protein Alternatives as Meat Extenders and Meat Analogs. Compr. Rev. Food Sci. Food Saf..

[22] Lombardi A., Vecchio R., Borrello M., Caracciolo F., Cembalo L. (2019). Willingness to pay for insect-based food: The role of information and carrier. Food Qual. Prefer..

[23] Verbeke W. (2015). Profiling consumers who are ready to adopt insects as a meat substitute in a Western society. Food Qual. Prefer..

[24] Post M.J., Levenberg S., Kaplan D.L., Genovese N., Fu J., Bryant C.J., Negowetti N., Verzijden K., Moutsatsou P. (2020). Scientific, sustainability and regulatory challenges of cultured meat. Nat. Food.

[25] Hocquette J.F. (2016). Is in vitro meat the solution for the future?. Meat Sci..

[26] Tuomisto H.L., Teixeira de Mattos M.J. (2011). Environmental Impacts of Cultured Meat Production. Environ. Sci. Technol..

[27] Lynch J., Pierrehumbert R. (2019). Climate Impacts of Cultured Meat and Beef Cattle. Front. Sustain. Food Syst..

[28] Mattick C.S., Landis A.E., Allenby B.R., Genovese N.J. (2015). Anticipatory Life Cycle Analysis of In Vitro Biomass Cultivation for Cultured Meat Production in the United States. Environ. Sci. Technol..

[29] Mancini M.C., Antonioli F. (2019). Exploring consumers’ attitude towards cultured meat in Italy. Meat Sci..

[30] Chriki S., Hocquette J.-F. (2020). The Myth of Cultured Meat: A Review. Front. Nutr..

[31] Muralikrishna I.V., Manickam V. (2017). Life Cycle Assessment. Environ. Manag..

[32] Wu Y., Su D. (2020). Review of Life Cycle Impact Assessment (LCIA) Methods and Inventory Databases. Sustain. Prod. Dev..

[33] Scharf A., Breitmayer E., Carus M. (2019). Review and Gap-Analysis of LCA-Studies of Cultured Meat for the Good Food Institute. www.nova-institut.eu.

[34] Smetana S., Mathys A., Knoch A., Heinz V. (2015). Meat alternatives: Life cycle assessment of most known meat substitutes. Int. J. Life Cycle Assess..

[35] Tuomisto H., Ellis M., Haastrup P., Schenck R., Huizenga D. (2014). Environmental impacts of cultured meat: Alternative production scenarios. Proceedings of the 9th International Conference on Life Cycle Assessment in the Agri-Food Sector.

[36] Arvidsson R., Tillman A.-M., Sandén B.A., Janssen M., Nordelöf A., Kushnir D., Molander S. (2018). Environmental Assessment of Emerging Technologies: Recommendations for Prospective LCA. J. Ind. Ecol..

[37] Sinke P., Odegard I. (2021). LCA of Cultivated Meat Future Projections for Different Scenarios. www.cedelft.eu.

[38] Williams A., Audsley E., Sandars D.L. (2006). Determining the Environmental Burdens and Resource Use in the Production of Agricultural and Horticultural Commodities. www.silsoe.cranfield.ac.uk.

[39] European Comission (2010). JRC Publications Repository-International Reference Life Cycle Data System (ILCD) Handbook-General guide for Life Cycle Assessment-Provisions and Action Steps. https://publications.jrc.ec.europa.eu/repository/handle/JRC58190.

[40] Fraeye I., Kratka M., Vandenburgh H., Thorrez L. (2020). Sensorial and Nutritional Aspects of Cultured Meat in Comparison to Traditional Meat: Much to Be Inferred. Front. Nutr..

[41] Good Food Institute (2021). Cultivated Meat Cell Culture Media|Deep Dive|GFI. https://gfi.org/science/the-science-of-cultivated-meat/deep-dive-cultivated-meat-cell-culture-media/.

[42] D’Este M., Alvarado-Morales M., Angelidaki I. (2018). Amino acids production focusing on fermentation technologies—A review. Biotechnol. Adv..

[43] Wang Y., Ouyang F. (1999). Recycle of Cytodex-3 in Vero cell culture. Bioprocess Eng..

[44] Moritz M.S.M., Verbruggen S.E.L., Post M.J. (2015). Alternatives for large-scale production of cultured beef: A review. J. Integr. Agric..

[45] Post M.J. (2014). Cultured beef: Medical technology to produce food. J. Sci. Food Agric..

[46] Stephens N., Di Silvio L., Dunsford I., Ellis M., Glencross A., Sexton A. (2018). Bringing cultured meat to market: Technical, socio-political, and regulatory challenges in cellular agriculture. Trends Food Sci. Technol..

[47] De Schauwer C., Meyer E., Van de Walle G.R., Van Soom A. (2011). Markers of stemness in equine mesenchymal stem cells: A plea for uniformity. Theriogenology.

[48] Ulloa-Montoya F., Verfaillie C.M., Hu W.S. (2005). Culture systems for pluripotent stem cells. J. Biosci. Bioeng..

[49] Bogliotti Y.S., Wu J., Vilariño M., Suzuki K., Belmonte J.C., Ross P.J. (2017). 2 Bovine Embryonic Stem-Like Cells Derived from in Vitro-Produced Blastocysts. Reprod. Fertil. Dev..

[50] Kadim I.T., Mahgoub O., Baqir S., Faye B., Purchas R. (2015). Cultured meat from muscle stem cells: A review of challenges and prospects. J. Integr. Agric..

[51] Hill A.B.T., Bressan F.F., Murphy B.D., Garcia J.M. (2019). Applications of mesenchymal stem cell technology in bovine species. Stem Cell Res. Ther..

[52] Ben-Arye T., Levenberg S. (2019). Tissue Engineering for Clean Meat Production. Front. Sustain. Food Syst..

[53] Zhang G., Zhao X., Li X., Du G., Zhou J., Chen J. (2020). Challenges and possibilities for bio-manufacturing cultured meat. Trends Food Sci. Technol..

[54] Verbruggen S., Luining D., van Essen A., Post M.J. (2017). Bovine myoblast cell production in a microcarriers-based system. Cytotechnology.

[55] Zhang M., Li L., Bai J. (2020). Consumer acceptance of cultured meat in urban areas of three cities in China. Food Control.

[56] Wilfart A., Gac A., Salaün Y., Aubin J., Espagnol S. (2021). Allocation in the LCA of meat products: Is agreement possible?. Clean. Environ. Syst..

[57] de Vries M., de Boer I.J.M. (2010). Comparing environmental impacts for livestock products: A review of life cycle assessments. Livest. Sci..

[58] Marti D.L., Johnson R.J., Mathews K.H. (2012). Where’s the (not) meat? By products from beef and pork production. J. Curr. Issues Glob..

[59] Romano E., Roma R., Tidona F., Giraffa G., Bragaglio A. (2021). Dairy farms and life cycle assessment (LCA): The allocation criterion useful to estimate undesirable products. Sustainability.

[60] Yan M.J., Humphreys J., Holden N.M. (2011). An evaluation of life cycle assessment of European milk production. J. Environ. Manag..

[61] Yu C., Penn L.D., Hollembaek J., Li W., Cohen L.H. (2004). Enzymatic tissue digestion as an alternative sample preparation approach for quantitative analysis using liquid chromatography-tandem mass spectrometry. Anal. Chem..

[62] Lee S.Y., Kang H.J., Lee D.Y., Kang J.H., Ramani S., Park S., Hur S.J. (2021). Principal protocols for the processing of cultured meat. J. Anim. Sci. Technol..

[63] Hendijani F. (2017). Explant culture: An advantageous method for isolation of mesenchymal stem cells from human tissues. Cell Prolif..

[64] Ding S., Swennen G.N.M., Messmer T., Gagliardi M., Molin D.G.M., Li C., Zhou G., Post M.J. (2018). Maintaining bovine satellite cells stemness through p38 pathway. Sci. Rep..

[65] Jang T.H., Park S.C., Yang J.H., Kim J.Y., Seok J.H., Park U.S., Choi C.W., Lee S.R., Han J. (2017). Cryopreservation and its clinical applications. Integr. Med. Res..

[66] Freshney R.I., Capes-Davis A., Gregory C., Przyborski S. (2015). Culture of Animal Cells: A Manual of Basic Technique and Specialized Applications.

[67] Seah J.S.H., Singh S., Tan L.P., Choudhury D. (2021). Scaffolds for the manufacture of cultured meat. Crit. Rev. Biotechnol..

[68] Allan S.J., De Bank P.A., Ellis M.J. (2019). Bioprocess Design Considerations for Cultured Meat Production with a Focus on the Expansion Bioreactor. Front. Sustain. Food Syst..

[69] O’Brien F.J. (2011). Biomaterials & scaffolds for tissue engineering. Mater. Today.

[70] Ben-Arye T., Shandalov Y., Ben-Shaul S., Landau S., Zagury Y., Ianovici I., Lavon N., Levenberg S. (2020). Textured soy protein scaffolds enable the generation of three-dimensional bovine skeletal muscle tissue for cell-based meat. Nat. Food.

[71] Swartz E. (2019). SBE Special Section: Industrial Biotechnology. www.aiche.org/cep.

[72] Eagle H. (1959). Amino acid metabolism in mammalian cell cultures. Science.

[73] van der Valk J., Brunner D., De Smet K., Svenningsen Å.F., Honegger P., Knudsen L.E., Lindl T., Noraberg J., Price A., Scarino M.L. (2010). Optimization of chemically defined cell culture media-Replacing fetal bovine serum in mammalian in vitro methods. Toxicol. Vitr..

[74] O’Neill E.N., Cosenza Z.A., Baar K., Block D.E. (2021). Considerations for the development of cost-effective cell culture media for cultivated meat production. Compr. Rev. Food Sci. Food Saf..

[75] Schnellbaecher A., Binder D., Bellmaine S., Zimmer A. (2019). Vitamins in cell culture media: Stability and stabilization strategies. Biotechnol. Bioeng..

[76] Yuan P., Cui S., Liu Y., Li J., Du G., Liu L. (2020). Metabolic engineering for the production of fat-soluble vitamins: Advances and perspectives. Appl. Microbiol. Biotechnol..

[77] Wang Y., Liu L., Jin Z., Zhang D. (2021). Microbial Cell Factories for Green Production of Vitamins. Front. Bioeng. Biotechnol..

[78] Salim I., González-García S., Feijoo G., Moreira M.T. (2019). Assessing the environmental sustainability of glucose from wheat as a fermentation feedstock. J. Environ. Manag..

[79] Zheng X., Baker H., Hancock W.S., Fawaz F., McCaman M., Pungor E. (2006). Proteomic analysis for the assessment of different lots of fetal bovine serum as a raw material for cell culture. Part IV. Application of proteomics to the manufacture of biological drugs. Biotechnol. Prog..

[80] Overton T.W. (2014). Recombinant protein production in bacterial hosts. Drug Discov. Today.

[81] Kim Y.S., Lee H.-J., Han M., Yoon N., Kim Y., Ahn J. (2021). Effective production of human growth factors in Escherichia coli by fusing with small protein 6HFh8. Microb. Cell Fact..

[82] Taha M., Silva F., Quental M.J., Ventura S., Freire M., Coutinho J.A.P. (2014). Good’s buffers as a basis for developing self-buffering and biocompatible ionic liquids for biological research. Green Chem..

[83] Specht L. (2020). GFI.ORG Creating a healthy, humane, and sustainable food supply. An Analysis of Culture Medium Costs and Production Volumes for Cultivated Meat.

[84] Bilal M., Mehmood S., Rasheed T., Iqbal H.M.N. (2020). Antibiotics traces in the aquatic environment: Persistence and adverse environmental impact. Curr. Opin. Environ. Sci. Health.

[85] Kumar K., Gupta S.C., Chander Y., Singh A.K. (2005). Antibiotic Use in Agriculture and Its Impact on the Terrestrial Environment. Adv. Agron..

[86] Stephanopoulos G.N., Aristidou A.A., Nielsen J. (1998). Examples of Pathway Manipulations: Metabolic Engineering in Practice. Metab. Eng..

[87] Mohan M. (2018). Perovskite Photovoltaics: Life Cycle Assessment. Perovskite Photovolt. Basic Adv. Concepts Implement.

[88] Casey J.W., Holden N.M. (2006). Quantification of GHG emissions from sucker-beef production in Ireland. Agric. Syst..

[89] Kumm K.I. (2002). Sustainability of organic meat production under Swedish conditions. Agric. Ecosyst. Environ..

[90] Nijdam D., Rood T., Westhoek H. (2012). The price of protein: Review of land use and carbon footprints from life cycle assessments of animal food products and their substitutes. Food Policy.

[91] Sonesson U., Davis J., Flysjö A., Gustavsson J., Witthöft C. (2017). Protein quality as functional unit–A methodological framework for inclusion in life cycle assessment of food. J. Clean. Prod..

[92] Jiménez-Colmenero F., Carballo J., Cofrades S. (2001). Healthier meat and meat products: Their role as functional foods. Meat Sci..

[93] Drewnowski A., Rehm C.D., Martin A., Verger E.O., Voinnesson M., Imbert P. (2015). Energy and nutrient density of foods in relation to their carbon footprint. Am. J. Clin. Nutr..

[94] Schau E.M., Fet A.M. (2007). LCA studies of food products as background for environmental product declarations. Int. J. Life Cycle Assess..

[95] Heller M.C., Keoleian G.A., Willett W.C. (2013). Toward a Life Cycle-Based, Diet-level Framework for Food Environmental Impact and Nutritional Quality Assessment: A Critical Review. Environ. Sci. Technol..

[96] Villares M., Işıldar A., van der Giesen C., Guinée J. (2017). Does ex ante application enhance the usefulness of LCA? A case study on an emerging technology for metal recovery from e-waste. Int. J. Life Cycle Assess..

[97] Gavankar S., Suh S., Keller A.A. (2015). The Role of Scale and Technology Maturity in Life Cycle Assessment of Emerging Technologies: A Case Study on Carbon Nanotubes. J. Ind. Ecol..

[98] van der Giesen C., Cucurachi S., Guinée J., Kramer G.J., Tukker A. (2020). A critical view on the current application of LCA for new technologies and recommendations for improved practice. J. Clean. Prod..

[99] Notarnicola B., Sala S., Anton A., McLaren S.J., Saouter E., Sonesson U. (2017). The role of life cycle assessment in supporting sustainable agri-food systems: A review of the challenges. J. Clean. Prod..

[100] Gac A., Salou T., Espagnol S., Ponchant P., Dollé J.-B., Van Der Werf H.M.G. (2014). An Original Way of Handling Co-Products with a Biophysical Approach in LCAs of Livestock Systems. www.ademe.fr/agribalyse-en.

[101] Sellitto M.A., Hermann F.F. (2016). Prioritization of green practices in GSCM: Case study with companies of the peach industry. Gestão Produção.

